# “I didn’t even wonder why I was on the floor” – mixed methods exploration of stroke awareness and help-seeking behaviour at stroke symptom onset

**DOI:** 10.1186/s12913-024-11276-6

**Published:** 2024-08-02

**Authors:** Loraine Busetto, Christina Stang, Franziska Herzog, Melek Sert, Johanna Hoffmann, Jan Purrucker, Fatih Seker, Martin Bendszus, Wolfgang Wick, Matthias Ungerer, Christoph Gumbinger

**Affiliations:** 1 Department of Neurology, Heidelberg University Hospital, Im Neuenheimer Feld 400, 69120 Heidelberg, Germany; 2 Institute of Medical Virology, Goethe University Frankfurt, University Hospital, Paul-Ehrlich-Str. 40, 60590 Frankfurt am Main, Germany; 3 Department of Paraplegia, Heidelberg University Hospital, Schlierbacher Landstraße 200a, 69118 Heidelberg, Germany; 4 Department of Neuroradiology, Heidelberg University Hospital, Im Neuenheimer Feld 400, 69120 Heidelberg, Germany

**Keywords:** All clinical neurology, Medical care, Stroke awareness, Help-seeking behaviour, Mixed methods research, Qualitative research, Acute stroke

## Abstract

**Introduction:**

To better target stroke awareness efforts (pre and post first stroke) and thereby decrease the time window for help-seeking, this study aims to assess quantitatively *whether* stroke awareness is associated with appropriate help-seeking at symptom onset, and to investigate qualitatively *why* this may (not) be the case.

**Methods:**

This study conducted in a German regional stroke network comprises a convergent quantitative-dominant, hypothesis-driven mixed methods design including 462 quantitative patient questionnaires combined with qualitative interviews with 28 patients and seven relatives. Quantitative associations were identified using Pearson’s correlation analysis. Open coding was performed on interview transcripts before the quantitative results were used to further focus qualitative analysis. Joint display analysis was conducted to mix data strands. Cooperation with the Patient Council of the Department of Neurology ensured patient involvement in the study.

**Results:**

Our hypothesis that stroke awareness would be associated with appropriate help-seeking behaviour at stroke symptom onset was partially supported by the quantitative data, i.e. showing associations between some dimensions of stroke awareness and appropriate help-seeking, but not others. For example, knowing stroke symptoms is correlated with recognising one’s own symptoms as stroke (*r* = 0.101; *p* = 0.030*; *N* = 459) but not with no hesitation before calling help (*r* = 0.003; *p* = 0.941; *N* = 457). A previous stroke also makes it more likely to recognise one’s own symptoms as stroke (*r* = 0.114; *p* = 0.015*; *N* = 459), but not to be transported by emergency ambulance (*r* = 0.08; *p* = 0.872; *N* = 462) or to arrive at the hospital on time (*r* = 0.02; *p* = 0.677; *N* = 459). Qualitative results showed concordance, discordance or provided potential explanations for quantitative findings. For example, qualitative data showed processes of denial on the part of patients and the important role of relatives in initiating appropriate help-seeking behaviour on patients’ behalf.

**Conclusions:**

Our study provides insights into the complexities of the decision-making process at stroke symptom onset. As our findings suggest processes of denial and inabilities to translate abstract disease knowledge into correct actions, we recommend to address relatives as potential saviours of loved ones, increased use of specific situational examples (e.g. lying on the bathroom floor) and the involvement of patient representatives in the preparation of informational resources and campaigns. Future research should include mixed methods research from one sample and more attention to potential reporting inconsistencies.

## Introduction

Acute ischemic stroke is one of the leading causes of death and acquired disability worldwide. Acute treatment options include stroke unit treatment, intravenous thrombolysis (IVT) and endovascular thrombectomy (EVT), all with strongly time-dependent treatment effects. While institutional and regulatory efforts have addressed the time frames from emergency call to treatment initiation [1–5], the time from symptom onset to first help-seeking is largely determined by decisions made by individual medical laypeople. Efforts for raising awareness of stroke are usually based on the assumption that increased stroke awareness will contribute to an increased likelihood of patients behaving correctly, and thereby an increased likelihood of timely treatment access.

However, a positive effect of these efforts has not been shown consistently [4, 6–15]. Moreover, evaluations use a wide range of outcome measures, including knowledge of risk factors, symptoms and treatments [9–14], action taken [9], emergency department visits [8], thrombolysis rates [8], initiation of reperfusion therapy [15] or functional outcome at discharge [7] – all capturing different aspects of how well a person is informed about stroke, knows what to do or actually implements the recommended action. This means that it is not clear to what extent knowledge of stroke symptoms can actually predict good health outcomes, or whether timely presentation to emergency services can really be attributed to higher stroke awareness. Several qualitative studies have pointed out the complexity of the decision-making-process, which in addition to patient-specific factors, is also subject to outside influences [16–18].

This study aims to (1) assess quantitatively *whether* different aspects of stroke awareness were associated with appropriate help-seeking behaviour at stroke symptom onset, and to (2) investigate qualitatively *why* this may (not) have been the case. We expect our results to help inform outreach campaigns and awareness efforts to better reach its target groups and intended goals for improved stroke outcomes.

## Methods

### Mixed methods research design

**Fig. 1:**
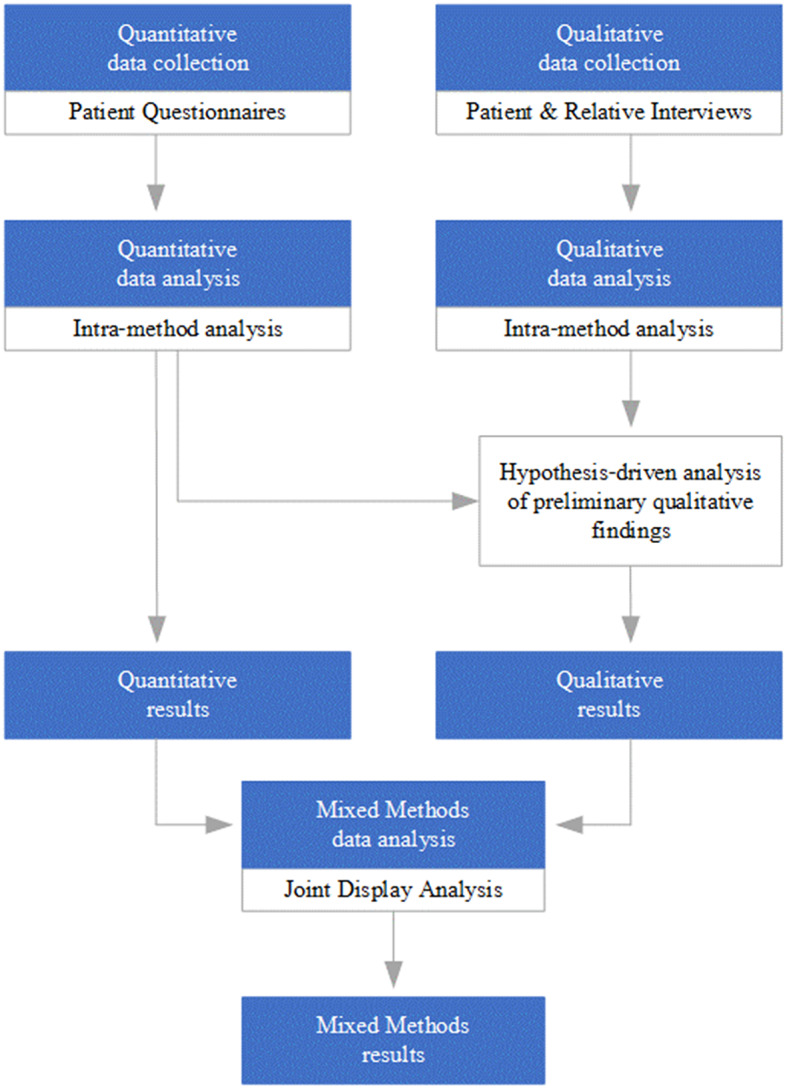
Mixed methods research design

This study used a convergent quantitative-dominant, hypothesis-driven mixed methods design including patient questionnaires and semi-structured interviews with patients and relatives (Fig. 1). The theoretical framework is informed by the COMIC Model, developed for the evaluation of complex care interventions, such as stroke care provision. It focuses on aspects beyond the medical (such as patient-centeredness) and specifically considers the context in which an intervention is implemented, as needed for the current study [19]. The study was conducted in a German regional stroke network (FAST; www.fast-schlaganfall.de). Ethics approval was obtained from the Medical Faculty of Heidelberg University (S-306/2016; S-682/2017). All study participants provided written informed consent. We report our findings in line with applicable standards [20].

The mixed methods integration strategy was to compare (especially regarding patient data) and to expand (especially regarding relative data) [21]. The mixed methods research data inventory [21] is shown in Table 1. We hypothesised that stroke awareness would be associated with appropriate help-seeking behaviour at stroke symptom onset. We defined “stroke awareness” as having information about stroke before the stroke occurred using the concepts “knowing stroke symptoms”, “familiarity with information campaigns”, “having experienced one or more previous strokes” and “knowing other stroke patients” or “having discussed stroke symptoms with other stroke patients”. We defined appropriate help-seeking as responding to a suspected stroke by seeking the appropriate help immediately upon symptom onset, measured using the concepts “recognising the symptoms as stroke”, “no hesitation before calling for help”, “transportation to hospital by emergency ambulance”, and “arrival at hospital within the 4.5 h therapeutic time window”. For mixing of data strands, we conducted a joint display analysis to assess for “fit” and draw meta-inferences according to the categories of concordance, expansion, complementarity or discordance of quantitative and qualitative findings which are addressed in the Discussion [22, 23].

**Table 1:** Details of the data collection and intra-method data analysis for the quantitative patient questionnaires and the qualitative patient and relative questionnaires (see also references [24–26])

	Questionnaires	Interviews
***Data collection***		
Inclusion criteria:	Patients admitted with stroke (including transient ischaemic attack) as their main hospital admission diagnosis were eligible for participation.	The sampling strategy was part of the larger qualitative “FAST” study, which aimed to evaluate different aspects of acute stroke treatment provision from the perspective of staff, patients, and relatives [24, 25]. Specific sub-study foci included telestroke provision, patient-centredness in the hyper-acute treatment phase, and the description of stroke patient pathways, in addition to this study’s focus on the pre-hospital phase of the patient journey. Within the FAST study, we used a purposive sampling strategy to recruit interviewees from different professional groups for staff interviewees (not reported in this sub-study) and with different stroke pathway experiences for the patient and relative interviews. This included, for example, experiences regarding transfer modes to hospital (helicopter or ambulance, drip-and-ship or mothership), telestroke, treatment at more or less specialised hospitals as well as different treatment and health outcomes.
Exclusion criteria:	Exclusion criteria were the inability to provide informed consent, e.g. due to clinical conditions, including aphasia or severe stroke, dementia, delirium, language barrier, discharge within 24 h, death or no provision of informed consent.	Exclusion criteria were lack of informed consent, age under 18 years and insufficient language skills (in German).
Recruitment:	The quantitative sub-study included patients from a university hospital and from a primary stroke center in a rural region. For the questionnaire, patients were recruited consecutively over a period of 6 months, starting in January 2017.	Recruitment and data collection for the qualitative interviews took place from May to July 2018 at Heidelberg University hospital and from July to September 2019 at two primary stroke centers.
Study participation:	All patients provided written consent at the time of admission, and the survey was performed on the following day. The questionnaire was provided in German and consisted of two parts, with the first completed by patients with minimal assistance by hospital staff when required, and the second part by the treating physician.	Interviews were conducted in German, approximately one month after stroke. A semi-structured interview guide based on the COMIC Model was used and piloted with a stroke self-help group. Audio-recordings were transcribed verbatim.
Research team	Not applicable	The core team of four researchers was responsible for data collection and analysis and not involved in patient care: LB is a social scientist experienced in qualitative research who supervised four Master students in health services research with backgrounds in nursing (JH), speech therapy (CS), physical therapy (FH), and health management (MS).
***Data analysis***		
Intra-method analysis	For the quantitative intra-method analysis, patient characteristics were described using standard descriptive statistics. Associations were identified using Pearson’s correlation analysis. Strength of association was indicated by the Pearson correlation coefficient (Pearson’s r). P-values < 0.05 (two-sided) were considered to be statistically significant. Statistical analyses were conducted with SPSS 25.0 (IBM Corp., Armonk, NY).	For qualitative intra-method analysis, interview transcripts were coded by at least two researchers using MaxQDA-software (2018, VERBI, Berlin, Germany). Using more than one coder makes it less likely that important findings are overlooked or interpreted in only one specific way. The coding process was informed by the theoretical framework but open to new findings, followed by axial and selective coding. In regular consultations within the research team, the coding process and scheme were discussed and adapted where necessary. After coding of all transcripts was completed, the quantitative results were used to focus the qualitative analysis on the aspects of stroke awareness and help-seeking behaviour as outlined for the questionnaires. Quotes from interviews were selected and translated from German to English by the research team.

### Data collection and analysis

Quantitative and qualitative data were collected separately. The quantitative data collection consisted of a questionnaire for patients admitted with acute stroke at an urban university hospital or a rural primary stroke center. Patients were recruited consecutively over a period of 6 months, starting in January 2017. The questionnaires were completed on the day after admission by the patients and their treating physician. Quantitative data were analysed using standard descriptive statistics. Associations were identified using Pearson’s correlation analysis. More detailed information on the quantitative questionnaire is published elsewhere [26].

For the qualitative data collection, semi-structured interviews were conducted with stroke patients and their relatives. A purposive sampling strategy was used to include interviewees with different stroke pathway experiences such as different transfer modes (helicopter or ambulance), admission at more or less specialised hospitals as well as different health outcomes. Recruitment and data collection place from May to July 2018 at Heidelberg University hospital and from July to September 2019 at two primary stroke centers. Interviews were conducted in German, approximately one month after stroke. The interview guide was piloted in advance with members of a regional stroke self-help group. For qualitative intra-method analysis, interview transcripts were coded by at least two researchers using MaxQDA-software (2018, VERBI, Berlin, Germany). After coding of all transcripts was completed, the quantitative results were used to focus the qualitative analysis on the aspects of stroke awareness and help-seeking behaviour as outlined for the questionnaires.

More detailed information on the respective methods of data collection and intra-method data analysis are shown in Table 2.

**Table 2:** Mixed methods data inventory

Matched constructs:	
**Quantitative patient questionnaire**	Qualitative topic guide for patient and relative interviews
**Dimensions of stroke awareness:**	
• Which symptoms would you associate with a stroke [Multiple answers possible! ]? (-> correct identification of at least 5 out of 8 symptoms indicative of stroke)	• Did you have information about the disease stroke before you experienced your own stroke? (Sources? ) • *Information brought up by respondents when discussing what happened and how they reacted at symptom onset*
• Have you heard of educational/information campaigns about stroke? (-> Yes / No)	• Did you have information about the disease stroke before you experienced your own stroke? (Sources? )
• Preexisting medical conditions -> Previous stroke (-> Yes / No)	• Was this your first stroke? • Were you aware that you would/could be at risk for stroke?
• Has a family member / friend / neighbor of yours suffered a stroke? (-> Yes / No) • If yes: Did you discuss the stroke symptoms with this person? (-> Yes / No)	• Do/did you know other stroke patients (before/after your own stroke)? To what extent do you think their experiences are comparable or different to yours? • *Information brought up by respondents when discussing why they had been able to recognise their symptoms as stroke*,* based on other people’s experiences*
**Dimensions of help-seeking behaviour:**	
• Did you interpret your symptoms to be indicative of a stroke? (-> Yes / No)	• Can you tell me about how it all began? The first symptoms of your stroke? • Who noticed when and how that something was not right?
• Did you hesitate before you contacted emergency services? (-> Yes / No)	• Who noticed when and how that something was not right? • Who reacted when and how? (e.g. by alerting emergency services / contacting GP / going to hospital on your own) • *For ethical reasons*,* we did not explicitly address whether they had reacted too late*
• Time to arrival at hospital after first symptoms: _______min (-> ≤4.5 h vs. >4.5 h	• Can you tell me about how it all began? The first symptoms of your stroke? • Who noticed when and how that something was not right? • Who reacted when and how? (e.g. by alerting emergency services / contacting GP / going to hospital on your own) • Can you remember how you were transported to hospital? • If patient went to hospital on their own: Did you contact your GP beforehand? How did you get to hospital (e.g. who drove you)? Which reasons impacted on your choice of hospital? • *Information interpreted from above timeline. For ethical reasons*,* we did not explicitly address whether they had arrived too late*
• How did you reach the hospital? (-> Hospitalisation by emergency medical services)	• Who reacted when and how? (e.g. by alerting emergency services / contacting GP / going to hospital on your own) • Can you remember how you were transported to hospital? • If patient went to hospital on their own: Did you contact your GP beforehand? How did you get to hospital (e.g. who drove you)? Which reasons impacted on your choice of hospital?

### Patient and public involvement

A stroke self-help group consulted on the qualitative design and helped pilot the interviews. Stakeholder validation of preliminary results was conducted with the Patient Council of the Department of Neurology on 17 November 2020, which showed agreement with findings outside the study sample and provided insights into discordance between quantitative and qualitative findings (see Discussion).

## Results

### Baseline characteristics (questionnaires)

In total, 462 patients were included in the quantitative analysis. Median age was 71.5 years (IQR: 60–79) and 47.4% of patients were female. Median premorbid Rankin scale (pmRS) was 0 (0–2). Other baseline characeristics including primary admission hospital, health status and risk factors are reported in Table 3.

**Table 3:** Baseline characteristics (quantitative sample)

Characteristic	Patients
Number of patients, n	462
Age, median (IQR)	72 (60–79)
Sex female, n (%)	219 (47.4)
Admitted to EVT-capable urban hospital, n (%)	344 (74.5)
pmRS, median (IQR)	0 (0–2)
mRS at admission, median (IQR)	2 (1–3)
NIHSS at admission, median (IQR)	2 (1–5)
Cardiovascular risk factors	
- Arterial Hypertension, n (%)	348 (75.3)
- Hypercholesterolemia, n (%)	196 (42.4)
- Smoking, n (%)	129 (27.9)
- Atrial fibrillation, n (%)	98 (21.2)
- Diabetes mellitus, n (%)	93 (20.1)
- Recurrent stroke, n (%)	95 (20.6)

*Notes IQR* interquartile range, *mRS* morbid Rankin Scale, *NIHSS* National Institutes of Stroke Scale, *pmRS* premorbid Rankin scale, *SD* standard deviation

**Fig. 2:**
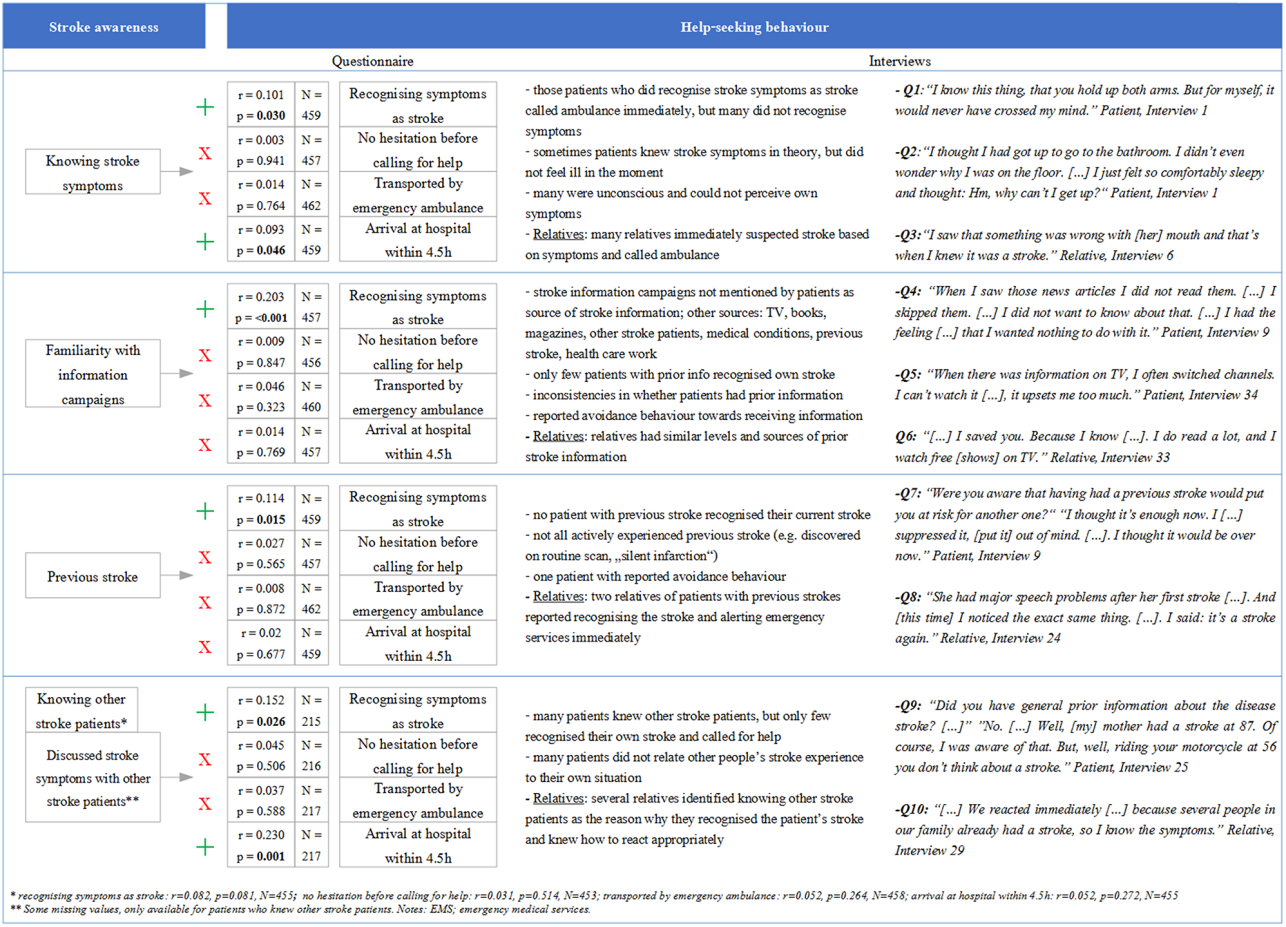
Summary of main findings

### Patient and relative characteristics (interviews)

We conducted 35 interviews, including 28 patient interviews and seven relative interviews. In 8 of the patient interviews, a relative was also present and occasionally participated. The interviews lasted between 20 and 82 min (median: 47 min, IQR: 32–59). Eleven patients were female (39%), and median age was 66 years (IQR: 60–78). Most patients had no prestroke disabilities as indicated by a pmRS of 0 (IQR 0–1). The mean NIHSS at admission was 8.7 (SD 7.7), indicating that most patients had not experienced a severe stroke. The primary admission hospital of eleven patients was an EVT-capable hospital; whereas the others were admitted at an IVT-capable hospital. Mean NIHSS at discharge was 2.6 (SD 2.6) while median mRS at discharge was 2 (IQR 1–3), showing a relatively good outcome after stroke. Of the seven relatives, six were female, and median age was 58, ranging from 23 to 72 years.

### Help-seeking and stroke awareness

Main findings are summarised in an integrated visual display in Fig. 2. This includes statistical results as well as qualitative interview quotes.

#### Knowing stroke symptoms

Questionnaires showed a positive correlation between knowing stroke symptoms and recognising symptoms as stroke (*N* = 459; *r* = 0.101; *p* = 0.030*) and arrival at hospital within 4.5 h (*N* = 459; *r* = 0.093; *p* = 0.046*), but not with no hesitation before calling for help (*N* = 457; *r* = 0.003; *p* = 0,941) and transportation by emergency ambulance (*N* = 462; *r* = 0.014; *p* = 0.764).

Five patient interviewees reported immediately knowing or strongly suspecting that they experienced a stroke. One recognized the stroke when he felt a sudden, strong stab of pain in the head and could not hold a water bottle. The other patient recognised the stroke when she saw her drooping cheek in the mirror. Of the five patients who recognized their stroke, four patients immediately called an ambulance or told their spouse to do so. The fifth patient was alone at home and could not physically react appropriately.

In contrast, eight patients who consciously experienced their symptoms stated that they had no idea it was a stroke, e.g. specifying that *“[it] was the last thing [he] would have thought of”* (Patient, Interview 12). These patients reported slurred speech, not being able to speak or answer questions, not being able to sit/stand/get up or walk (properly), not being able to use their leg(s), lying on the floor, and not being able to use their arm or hand (including dropping things). Another patient specified that even though she was aware of common stroke symptoms, she did not recognise them in her own case.

*I know this thing*,* that you hold up both arms. But for myself*,* it would never have crossed my mind*.

*Patient*,* Interview 1*

She and another patient emphasised that even though they consciously experienced one or more symptoms, they did not feel that something was wrong.

*I thought I had got up to go to the bathroom. I didn’t even wonder why I was on the floor. […] I just felt so comfortably sleepy and thought: Hm*,* why can’t I get up?*

*Patient*,* Interview 1*

Sometimes patients also initially attributed their symptoms to alternative explanations, i.e. an epileptic attack or hangover. Eight patients were unconscious or too confused to notice their symptoms or did not remember the situation. In these cases, other people called for help on their behalf. Twelve relatives present at symptom onset immediately knew or strongly suspected a stroke based on the symptoms, which included slurred speech, drooping mouth, not being able to speak, paresis, not being able to get up or walk properly, a cramped-up hand, and tingling feelings in one arm.

All relatives suspecting a stroke immediately called for help without waiting for the symptoms to improve or otherwise delaying the process.

*I saw that something was wrong with [her] mouth and that’s when I knew it was a stroke*.

*Relative*,* Interview 6*.

#### Familiarity with stroke information campaigns

Questionnaires showed a positive correlation between familiarity with stroke information campaigns and recognising symptoms as stroke (*r* = 0.203; *p* ≤ 0.001*; *N* = 457) but no correlation with no hesitation before calling for help (*r* = 0.009; *p* = 0.847; *N* = 456), transportation by emergency ambulance (*r* = 0.046; *p* = 0.323; *N* = 460), and arrival at hospital within 4.5 h (*r* = 0.014; *p* = 0.769; *N* = 457).

In the interviews, patients were asked about their prior knowledge about the disease stroke and if so, their information sources. Twelve patients indicated that they had had prior information about the disease stroke, naming information sources such as television shows, books and magazines on health topics, knowing other stroke patients, medical conditions because of which they had been told they were at risk for stroke, a previous (own) stroke, and working or volunteering in health care. Of these patients, two patients reported having recognised their stroke, both immediately asking their husbands to call help. Stroke information campaigns were not mentioned by the interviewees.

Many patients who answered “no” to the question “Did you have any prior information about the disease stroke?”, also reported knowing other stroke patients or having discussed their stroke risk or suspected stroke symptoms with a health professional in the months or years before their stroke. Two patients reported actively avoiding information on the topic

*When I saw those news articles*,* I did not read them. […] I skipped them. […] I did not want to know about that. […] I had the feeling […] that I wanted nothing to do with it.*

*Patient*,* Interview 9*

*When there was information on TV*,* I often switched channels. I can’t watch it […]*,* it upsets me too much.*

*Patient*,* Interview 34*

The latter patient is one of two patients who, despite indicating no prior information about stroke, recognized their stroke at symptom onset. The other patient reported that because of his regular check-up appointments for heart disease he was aware of his stroke risk. The patient was alone at home when the stroke happened but was found by a neighbour who immediately called an ambulance.

Only few patients who indicated having no prior information about stroke also reported not knowing any stroke patients and not having been aware that they were at risk of stroke. In these cases, it was the patient’s partner who initiated help-seeking. In one case, the patient’s wife called an ambulance because of the severity of the symptoms even though she did not realise it was a stroke at the time.

Nine relatives present at symptom onset said they had prior information about stroke, also citing television shows and books on health topics, knowing other stroke patients, the patient’s previous stroke, and volunteering in health care as their main information sources .

*Speaking to patient: I saved you. Because I know […]. I do read a lot*,* and I watch [shows] on TV*

*Relative*,* Interview 33*

All of these relatives recognised the patient’s stroke based on their symptoms and sought help immediately.

#### Previous stroke

Questionnaire data for having experienced one or more previous strokes showed a positive correlation with recognising symptoms as stroke (*r* = 0.114; *p* = 0.015*; *N* = 459) but no correlation with no hesitation before calling for help (*r* = 0.027; *p* = 0.565; *N* = 457), transportation by emergency ambulance (*r* = 0.008; *p* = 0.872; *N* = 462), and arrival at hospital within 4.5 h (*r* = 0.02; *p* = 0.677; *N* = 459).

In the qualitative patient sample, four patients had previously experienced a stroke. None of them recognised their second stroke, with two unconscious at symptom onset or unable to recall the situation later. In two cases, patients knew that a stroke had been discovered previously during a routine scan, but they had not been aware of it when it happened (so-called “silent infarctions”). A third patient had experienced his first stroke just a few weeks prior to his second while he was still in rehabilitation for the first. A fourth patient had experienced an acute stroke two years previously. This latter patient did not seem to (want to) realise that this would put him at risk for another stroke:

*Interviewer: “Were you aware that having had a previous stroke would put you at risk for another one?”*

*Interviewee: I thought it’s enough now. I […] suppressed it*,* [put it] out of my mind […]. I thought it would be over now.*

*Patient*,* Interview 9*

In one of the above cases, Patient 9’s wife recognized the stroke and alerted emergency services immediately. In the other cases, no relatives were present and emergency services were instead alerted by unrelated witnesses. A fifth case of a previous stroke was reported by the daughter of a stroke patient who was herself not included in this study. This patient had experienced a severe acute stroke approximately twelve years previously. The daughter reported this as the reason why she recognized her mother’s second stroke and called for help immediately:

*She had major speech problems after her first stroke […]. And [this time] I noticed the exact same thing. […] I said: it’s a stroke again.*

*Relative*,* Interview 24*

#### Knowing other stroke patients

Questionnaires showed no correlation between knowing other stroke patients and recognising symptoms as stroke (*r* = 0.082; *p* = 0.081; *N* = 455), no hesitation before calling for help (*r* = 0.031; *p* = 0.514; *N* = 453), transportation by emergency ambulance (*r* = 0.052; *p* = 0.264; *N* = 458), and arrival at hospital within 4.5 h (*r* = 0.052; *p* = 0.272; *N* = 455). For those patients who did know other stroke patients and who reported having discussed stroke symptoms with them, a positive correlation was found with recognising symptoms as stroke (*r* = 0.152; *p* = 0.026*; *N* = 215), and arrival at hospital within 4.5 h (*r* = 0.230; *p* = 0.001*; *N* = 217) but not with no hesitation before calling for help (*r* = 0.045; *p* = 0.506; *N* = 216) and transportation by emergency ambulance (*r* = 0.037; *p* = 0.588; *N* = 217).

In the interviews, thirteen patients reported knowing other stroke patients before, mostly family members and friends, but also colleagues, neighbours and acquaintances. Of these, two patients had recognised their own stroke and called for help immediately. One spoke in detail about her son-in-law’s stroke and thrombectomy treatment as well as the stroke experience of a friend, stating this as the reason *“[…] why [she and her husband] had known about stroke since then and also knew about the time window” (Patient*,* Interview 7)*. This was not the case for the other patient who first reported no prior information about stroke before mentioning that his mother had had one at a much older age:

*Interviewer: Did you have general prior information about the disease stroke?*

*Interviewee: No. […] Well*,* [my] mother had a stroke at [88]. Of course*,* I was aware of that. But*,* well*,* riding your motorcycle at [57]*,* you don’t think about a stroke*

*Patient*,* Interview 25*

A similar pattern was also visibile with other interviewees, who initially responded that they did not know other stroke patients before realising that this was not the case. Nine patients specifically stated that they did not know other stroke patients before their own stroke. Of these, three patients were able to recognise their own stroke, however citing other information sources such as check-ups for heart disease, working in health care, and TV programs.

Seven relatives present at symptom onset reported knowing other stroke patients, with several identifying this as the reason why they recognised their spouse’s stroke and responded appropriately.

*We reacted immediately […] because several people in our family already had a stroke*,* so I know the symptoms.*

*Relative*,* Interview 29*

## Discussion

We explored patients’ and relatives’ help-seeking behaviour at stroke symptom onset using quantitative questionnaires and qualitative interviews. Our hypothesis that having stroke awareness would be positively associated with appropriate help-seeking behaviour was partially supported by quantitative and qualitative data, which confirmed and contradicted each other and sometimes provided potential explanations for apparent inconsistencies, as we discuss below.

### Summary and discussion of main findings

Qualitative findings around the impact of *knowing stroke symptoms* were found to be partially in discordance with quantitative findings. Specifically, questionnaires showed patients with knowledge of stroke symptoms to be more likely to recognise their symptoms as stroke and to arrive at hospital on time. In contrast, interviews showed many patients to not have recognized their symptoms as stroke, even when they knew of common stroke symptoms. Two patients explained that they did not feel ill and even that they felt comfortable. This was confirmed by a former stroke patient in the Patient Council who reported not linking their general knowledge to their acute experience and inexplicably feeling safe and seeing everything through rose-tinted glasses. While the literature shows that lack of pain or perceived symptom severity can contribute to a diminished feeling of urgency, we were not able to find published descriptions of these feelings of comfort or safety [16, 27–29].

Regarding the importance of *familiarity with information campaigns*, our qualitative and quantitative findings complemented each other. While questionnaires showed that patients familiar with campaigns were more likely to recognise their stroke, interviewed patients reported other information sources. Findings from the published literature show a variety of results in terms the impact of stroke information campaigns, e.g. reporting (partial) effectiveness [7, 8, 10] but also rather limited impact [6, 9]. Notably, in our study, patient reporting of prior stroke information sometimes appeared inconsistent, e.g. when patients later spoke about a relative with stroke. This suggests that patients have better recall of some types of information than others [28]. It may also be suggestive of individual patient characteristics contributing to avoidance behaviour. Moloczij et al. called this the desire to “[maintain] a sense of normalcy”, describing several strategies used by patients to support their decision not to take any action, including denial, minimisation of symptoms, and compensating or adapting [16]. Wang et al. use descriptors such as “hesitating and puzzling” and “doubting – it may only be a minor problem” to describe this process experienced by stroke patients before initiating help-seeking [30].

Partial discordance was also found for *previous strokes*. While questionnaires showed patients with one or more previous strokes more likely to recognise their current symptoms as stroke, none of the five patients in the qualitative sample had recognised their current stroke. In their literature review of factors influence prehospital delay and stroke knowledge, Teuschl and Brainin (2010) find that only few studies report shorter time delays or better stroke knowledge in persons having suffered a previous stroke [27]. While silent (previous) infarctions may explain some of these instances, one patient who actively experienced their previous stroke reported avoidance behaviour before the second stroke. This was also reflected in Mackintosh et al.’s study of why people do (not) immediately contact emergency services, including several patients who recognised their second stroke but did not take action [28]. This observation was discussed in the Patient Council whose patient representatives showed surprise at the apparent lack of impact of previous stroke experiences. It was discussed whether stroke patients may not perceive themselves as living with a long-term condition requiring ongoing vigilance, but instead an isolated and completed incident.

Finally, qualitative and quantitative data were found to overlap and expand each other for *knowing other stroke patients* and *having discussed the disease stroke*. Interviews provided additional insights into possible reasons for when patients did *not* relate to others’ experiences and showed the importance of relatives knowing other stroke patients. Questionnaires showed no significant associations between knowing other stroke patients and the four dimensions of appropriate help-seeking behaviour, but patients who had *discussed* symptoms with other stroke patients were found to be more likely to recognise their stroke and to arrive at hospital on time. Again, there appeared to be inconsistencies in the interviews, with patients forgetting and then remembering knowing someone with stroke, and with many patients not relating others’ stroke experiences to their own situation. In contrast, several relatives identified knowing other stroke patients as the specific reason why they recognized the patient’s stroke and knew how to react. The importance of bystander involvement was explored by Mellon et al., identifying symptom recognition and help-seeking by witnesses as critical for a fast response [31]. For instance, Geffner et al. found that the decision to seek medical help was taken by patients in only 20.4% of cases [32]. Iverson et al. also found the presence of a bystander at symptom onset to be associated with appropriate help-seeking [15]. However, other qualitative findings are more nuanced, e.g. with Mc Sharry et al. reporting actions taken by others as having the potential to override patients’ own identification of symptoms and Moloczij et al. finding that sometimes the presence of another person contributed to delayed help-seeking, while at other times facilitating contact with medical services [16, 29]. In addition to patients’ and relatives’ own behaviour and decisions, studies also show the importance of system factors, such as inefficient pre-hospital triage for treatment delay [33].

### Strengths and limitations

As data collection was prepared and conducted independently, it was not always perfectly matched. One example of this is the fact that the rural-urban divide was not considered in detail in the qualitative data collection. This means that potentially important qualitative explanations of quantitative findings related to rural vs. urban differences were not explored in the current study, such as potential differences in information access, transport time or time-to-access to emergency services. Moreover, as is appropriate for qualitative interviews, prompting for more detailed information depended on the specific context and was therefore not feasible for all interviewees and all sub-questions. In the questionnaires, patients were asked about *prior* knowledge of stroke systems *after* they had their stroke. However, since it was completed on the day itself or day one after treatment, there would not have been much time for extended patient education. Additionally, the quantitative questionnaire was analysed with a pre-defined analysis plan and was collected over a (pre-defined) time period of six months. However, no power or sensitivity analysis was conducted in advance. Finally, our qualitative sample showed very good recovery, which probably affected the range of experiences and reactions covered in the interviews. One might assume that this overrepresentation of good outcomes could suggest a similar overrepresentation of study participants who “acted correctly”. However, given the importance of luck, bystander help, patients’ physical incapability to react and additional factors other than informed decision-making reported in this study, our results indicate that caution is warranted when interpreting good outcomes or arrival inside the time-window as proxies for having acted quickly or correctly (and vice versa). The main strengths of this study are its two-site design covering hospitals in urban and rural areas with differences in acute stroke treatment options, ensuring good external validity for Germany and countries covering larger geographical areas, its mixed methods approach allowing for integration of findings and generation of new perspectives of inquiry, and the involvement of patient representatives in the study preparation and conclusion.

### Recommendations

As quantitative and qualitative findings sometimes seemed contradictory, we recommend that future studies collect data from one patient sample (instead of two separate samples, as here), allowing for direct back-and-forth iterations.As qualitative interviews pointed towards relevant inconsistencies in patient reporting, e.g. of prior stroke knowledge even with regard to close family members, it might be worth re-examining the reliability of common quantitative measures of stroke awareness and help-seeking behaviour where these inconsistencies would remain hidden and potentially incorrect. Following the Patient Council discussions, future research may investigate the “comfortable lull” reported by two patients from the study sample and one patient from the Council. If found in more instances, this could contribute to patients not recognizing a situation as highly problematic and requiring urgent action. In terms of practice recommendations, a more family- or community-based approach to stroke information provision may be helpful, emphasising the opportunity to be a loved one’s saviour. This could lessen the impact of avoidance behaviour and increase the positive impact of the presence of a family member on the decision-making process. This may necessitate critical discussions of whether and how relatives should be able to override patient preferences for delayed or no help-seeking behaviour, especially when the patient’s capacity for decision-making is impaired. As many patients seemed unable to apply general knowledge of stroke symptoms in the acute situation, we suggest exploring an example-based approach to risk communication. Specific situational examples (e.g. lying on the floor in the middle of the night or falling down without knowing why) may be a more accessible source of information compared to paresis of the arms or legs. To provide this type of information in the most appropriate way to future patients and their relatives, it seems relevant to involve former stroke patients in the preparation and provision of these informational resources.

## Conclusion

Our study provides insights into the complexity of a decision-making process that is influenced by certain factors, but not others – e.g. a previous stroke makes it more likely that a patient recognises their symptoms as stroke, but not that they call for help without hesitation or arrive at the hospital on time. Interviews with patients and relatives provided in-depth insights into these seemingly contradictory findings, e.g. suggesting processes of denial or the inability to translate abstract knowledge into correct actions. We therefore recommend to address relatives as potential saviours of loved ones, increased use of specific situational examples (e.g. lying on the bathroom floor) and the involvement of patient representatives in the preparation of informational resources and campaigns.

## Data Availability

The datasets used and/or analysed during the current study are available from the corresponding author on reasonable request. This excludes interview transcripts as ethics requirements to ensure confidentiality do not allow for data sharing outside the research team.
